# Local indicators of geocoding accuracy (LIGA): theory and application

**DOI:** 10.1186/1476-072X-8-60

**Published:** 2009-10-28

**Authors:** Geoffrey M Jacquez, Robert Rommel

**Affiliations:** 1 BioMedware, Inc., 516 North State Street, Ann Arbor, MI, 48104-1236, USA; 2 Department of Environmental Health Sciences, The University of Michigan School of Public Health, Ann Arbor, MI, 48109-2029, USA

## Abstract

**Background:**

Although sources of positional error in geographic locations (e.g. geocoding error) used for describing and modeling spatial patterns are widely acknowledged, research on how such error impacts the statistical results has been limited. In this paper we explore techniques for quantifying the perturbability of spatial weights to different specifications of positional error.

**Results:**

We find that a family of curves describes the relationship between perturbability and positional error, and use these curves to evaluate sensitivity of alternative spatial weight specifications to positional error both globally (when all locations are considered simultaneously) and locally (to identify those locations that would benefit most from increased geocoding accuracy). We evaluate the approach in simulation studies, and demonstrate it using a case-control study of bladder cancer in south-eastern Michigan.

**Conclusion:**

Three results are significant. First, the shape of the probability distributions of positional error (e.g. circular, elliptical, cross) has little impact on the perturbability of spatial weights, which instead depends on the mean positional error. Second, our methodology allows researchers to evaluate the sensitivity of spatial statistics to positional accuracy for specific geographies. This has substantial practical implications since it makes possible routine sensitivity analysis of spatial statistics to positional error arising in geocoded street addresses, global positioning systems, LIDAR and other geographic data. Third, those locations with high perturbability (most sensitive to positional error) and high leverage (that contribute the most to the spatial weight being considered) will benefit the most from increased positional accuracy. These are rapidly identified using a new visualization tool we call the LIGA scatterplot.

Herein lies a paradox for spatial analysis: For a given level of positional error increasing sample density to more accurately follow the underlying population distribution increases perturbability and introduces error into the spatial weights matrix. In some studies positional error may not impact the statistical results, and in others it might invalidate the results. We therefore must understand the relationships between positional accuracy and the perturbability of the spatial weights in order to have confidence in a study's results.

## Background

Two aspects of geocoding accuracy are often of interest: completeness (e.g. how much of an address is present in the input to the geocoder itself; and what proportion of addresses are successfully geocoded) and positional accuracy (e.g. how closely the geocoded coordinates correspond to the true coordinates). This paper is concerned with positional accuracy; it does not seek to develop metrics for completeness. All geographic data whether arising from geocoding street addresses, global positioning systems, LIDAR and other devices have distributions of positional errors that arise during the data collection and coding process. This research develops an approach for identifying how sensitive spatial weight matrices are to positional error regardless of the data's origin or the data collection process. Geographic relationships are treated differently in different types of spatial models. Here we are concerned only with models that incorporate spatial weight matrices.

A major challenge in spatial statistical studies in general and of disease in particular is the need to undertake routine evaluation of the impact of positional accuracy on the results. In this paper we (1) develop a positional accuracy analysis approach that is generally applicable to a broad range of spatial and space-time statistics, (2) evaluate the performance of the approach in simulation studies, and (3) apply the new approach in a study of bladder cancer in Michigan.

Researchers often wish to measure characteristics of geographic patterns in data such as clustering, dispersion, and multivariate interactions. The data analyzed typically consist of observations that have a geographic location and time of observation in addition to the attributes of interest. For many human studies, the location is an address which is geocoded to convert it to a point on a projection space. Spatial weights are then calculated from these geocoded coordinates (e.g. distance between two points, adjacency, and nearest neighbor relationships) and used to calculate a spatial or space-time statistic. In this paper, we examine some of the ways in which geocoding accuracy can affect these families of spatial weights. We focus our inquiries on geocoded health data, but the approach we develop is generally applicable to data models that involve spatial coordinates with an associated positional error. The amount and distribution of positional error usually is not known in advance, and the approach we develop therefore uses simulation approaches to evaluate the perturbability of spatial weights to positional error.

A number of studies have shown that the geocoding process can introduce a substantial amount of error when compared with "ground-truthed" coordinates [1-3]. A recent review [4] of studies of geocoding reported mean positional errors from 58 to 96 meters in urban areas, and from 129 to 614 in rural settings (Table 1). Bonner et al. [1] reported mean geocoding errors of 96 meters in urban and 129 meters in rural settings. Cayo and Talbot [5] found mean errors of 58 m in urban and 614 meters in rural areas, and Ward et al. [3] reported mean positional errors of 77 meters in urban and 210 meters in rural settings. The maximum geocoding positional error observed in these studies was 18,742 meters, nearly 19 kilometers [5]. How might such error impact the results of spatial analyses?

**Table 1:** Empirical geocoding positional error distributions

	**Bonner et al. (2003)**	**Cayo & Talbot (2003)**	**Ward et al. (2005)**
**Urban/Town**			
Median	32	38	56
75^th ^percentile	-	-	92
90^th ^percentile	37	96	-
Mean	96	58	77
Max	1,551	1,088	687
**Rural/Nonurban**			
Median	52	201	88
75^th ^percentile	-	-	254
90^th ^percentile	61	1,544	-
Mean	129	614	210
Max	2,552	18,742	1,731

Summary of geocoding positional error (meters) from 3 studies. Source: Abe and Stinchcomb (2008).

Positional error propagation in environmental modeling has been an active research area for quite some time. Heuvelink [6] considered four techniques of location error propagation (pages 36-49): first and second-order Taylor expansions, Rosenblueths' method [7] and Monte Carlo simulation. These techniques provide mechanisms for propagating positional as well as attribute error in a broad variety of GIS-based models. Similarly, the assessment and modeling of positional accuracy itself has been extensively studied, providing alternative techniques for error modeling and for evaluating the precision, accuracy and bias of location-based data, see for example [8-10]. Nonetheless, research on how positional errors impact the spatial analysis of disease patterns has been limited [11], even though geocoding of health data is now standard practice in cancer and other disease registries [12]. Studies have demonstrated that geocoding error can have substantial impact on the results of spatial analyses of disease and that geographic bias may be expected whenever underlying risk factor(s) are associated with the probability of geocoding error [1,3,13]. The amount of bias depends on the extent that a study is rural, with bias higher in areas with small population densities [2,14]. Models have been developed for the shape of the probability distribution of geocoding errors [15], and for estimating critical parameters such as spatial intensity and risk [2,14,16,17]. Simulation studies of relationships between environmental exposures and health have demonstrated that the strength of the imputed odds relationship between exposures and health declined with decreasing geocoding accuracy [18]. Yet sensitivity analyses that quantify how spatial analyses depend on geocoding positional accuracy are not routinely undertaken in geographic disease studies, primarily because of the lack of theory, methods and tools. To date, simulation approaches are used in which a location error model is specified and the statistical power of a spatial analysis method under different error magnitudes is explored [19,20]. This is painstaking and time-consuming, and is not routinely accomplished. And while methods exist for substantially improving positional accuracy using manual and other approaches [21], these are labor-intensive and it makes sense to first determine whether improved accuracy is needed, and to prioritize locations that would benefit most from enhanced positional accuracy.

This paper develops techniques for quantifying the perturbability of spatial weights to geocoding accuracy. We find that a family of curves describes the relationship between positional error and perturbability, and demonstrate that these may be used to evaluate sensitivity of spatial analysis methods to positional accuracy in applied settings. Three results are significant. First, the shape of the probability distributions of positional error (e.g. circular, elliptical, cross) is not important; instead perturbability of spatial weights depends on the mean positional accuracy. Second, our methodology is general and allows researchers to evaluate the sensitivity of spatial statistics to positional error for specific geographies. Using our methodology, researchers may systematically evaluate the sensitivity of alternative spatial weight specifications to positional accuracy. Third, leverage analysis identifies those locations that contribute the most to the spatial statistic being considered. This information when coupled with knowledge of perturbability supports local analyses of sensitivity to positional error. Those locations with high perturbability and high leverage will benefit the most from increased positional accuracy. This important result allows researchers to identify those locations where positional error needs to be minimized without having to first visit those locations to quantify geocoding accuracy itself.

## Methods

This section presents details of the methods used for the perturbability analysis of weight matrices, the leverage analysis, and the LIGA scatterplot. We begin with an overview of the approach and a discussion of spatial weight matrices and specification of the alternative hypothesis.

We propose a four step approach to positional error sensitivity analysis.

Step 1: Evaluate sensitivity of alternative spatial weights to positional accuracy using perturbability analysis.

Step 2: Decide on the specification of the spatial weights (e.g. number of nearest neighbors to consider) based on the alternative hypothesis being considered and the results from Step 1. Specifically, select that spatial weight specification that most closely corresponds to the alternative hypothesis being considered and that is found to be least perturbable in Step 1.

Step 3: Use leverage analysis to quantify how much each location contributes to the spatial weight structure.

Step 4: Use the LIGA scatterplot of local perturbability vs. leverage to identify those locations most sensitive to positional accuracy and with the largest influence on the spatial weights.

### Spatial Weights and the Meaning of the Alternative Hypothesis

Step 2 above states "Decide on the specification of the spatial weights ... based on the alternative hypothesis being considered ...", but how does one determine the appropriate spatial weights given an alternative hypothesis? In practice, five considerations are important when specifying spatial weights.

#### 1. To reflect the spatial process

The spatial weights can be specified to quantify the alternative hypothesis or spatial process of interest [22,23], assuming of course one has some idea what that spatial process might be, and is able to articulate that process quantitatively. When working within a hypothesis-testing framework the spatial weights might describe a spatial "template" defining, for example, the shape and extent of the disease cluster(s) one would like to detect (e.g. an excess of disease risk within neighboring polygons). When used in spatial modelling, the spatial weights, or a set of spatial weights, define the spatial lags used to model spatial dependencies such as spatial autocorrelation, autocovariance and the semi-variogram. In that sense the spatial weights are constructed according to one's alternative hypotheses about spatial patterns - the disease cluster one wants to detect, or the spatial dependencies one wants to model.

#### 2. As a tool in knowledge discovery

Sometimes the researcher has only partial knowledge regarding the spatial process being explored - in fact one often undertakes a spatial study to better understand just what the underlying processes might be! When prior knowledge of the underlying spatial processes is lacking one can invoke the methods of Strong Inference originally proposed by Platt [24], who suggested one first construct the set of plausible hypotheses that might explain the observed data, and then undertake experiments (e.g. statistical tests) to systematically evaluate each hypothesis. The remaining hypothesis then is a plausible explanation for the observed spatial patterns. Often, the set of plausible hypotheses may change as the researcher works with the data and as new information comes to light.

#### 3. To assess scale dependency

When exploring sensitivity of the results to changes in spatial scale researchers may choose to use a set of different spatial weights corresponding to different spatial scales. For example, when exploring case-control clustering using Cuzick and Edwards' test the parameter *k*, the number of nearest neighbors to consider, may be varied by the researcher. The p-value of the test statistic, T_k_, tends to be minimized when *k *reflects the scale of case-clustering.

#### 4. As dictated by the data

Finally the specification of spatial weights depends on the type of data to be analyzed (e.g. points, lines, polygons) and their spatial resolution (e.g. census tracts, counties). It is very difficult to detect spatial processes occurring on finer scales than the scale of observation - e.g. to find a clustering of census tracts using county data.

#### 5. To minimize sensitivity to geocoding error

In this paper we suggest researchers routinely undertake an evaluation of the sensitivity of their spatial weight matrices to positional accuracy using the LIGA scatterplot.

We believe our approach has substantial practical implications since it makes possible routine sensitivity analysis of spatial weights (and hence the spatial statistics calculated from them) to positional accuracy.

### Perturbability of Weight Matrices

Our rationale is as follows: geocoding positional error impacts spatial and space-time statistics by changing the elements of the spatial or space-time weights matrix. Hence we must quantify how sensitive or "perturbable" a weight matrix is to positional accuracy in order to understand the impact of positional accuracy on any spatial statistic of interest.

We define approaches to quantifying perturbability under two assumptions. First, that the amount and distribution of geocoding positional error is unknown, in which case one wishes to quantify how different specifications of positional accuracy (e.g. functional form, mean and variance) impact the results. Second, that positional accuracy on average is the same across the locations and is not spatially structured. We later explore ways of relaxing this assumption to account, for example, for geocoding positional error being higher in rural areas.

Given *N *points in the geographic plane define a matrix of spatial or space-time weights, **W**, used in statistics of the form in Eqn 1:

(1)

Here *S *is a spatial statistic and **D **is a matrix of measures calculated from the attributes, which may include case-control identifiers, exposure metrics, and so on. Many inferential spatial statistics conform to this general form [25]. Examples include Cuzick and Edwards' test [26], Mantel's test [27], the Knox test [28] and the Vesta and Janus statistics [29], to name a few. Modeling approaches such as spatial regression, geostatistics [30], geographically-weighted regression [31] and others may also be written in this general form. The weights themselves may be nearest neighbor, adjacency or based on geographic distances (e.g. inverse distance squared), and our approach appears applicable to all of these.

We now develop perturbability analysis for nearest neighbor, adjacency and distance-based weights. Let **W **indicate the matrix of weights calculated from the "true" locations, and let **W' **indicate the weights after the locations have been perturbed by positional error. We then define a change matrix, **Δ**, whose elements are calculated as . Here *w*_*ij *_is the weight for locations *i *and *j *for the true locations and *w'*_*ij *_is the weight after it has been perturbed by positional error. Perturbability for the *i*^*th *^location is defined as the absolute value of the row sum of the i^th ^row of the change matrix plus the column sum of the i^th ^column of the change matrix:

(2)

A global measure of perturbability is the sum of the local perturbabilities:

(3)

Perturbability as defined in Equations 2 and 3 applies generally to all of the spatial and space-time weights we considered, including nearest neighbor, adjacency and distance-based weights, as summarized below. The application study later in this paper uses nearest-neighbor based local and global perturbability.

#### Nearest neighbor weights

**Figure 1:**
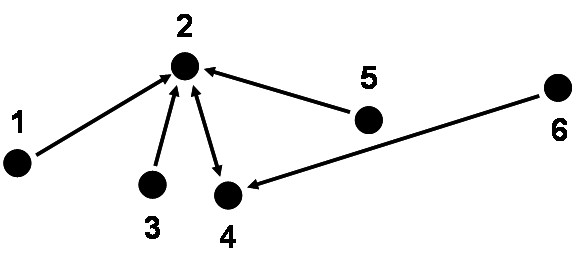
**Second-order (*k *= 2) nearest neighbor relationships for a set of six point locations**. Arrows indicate point-pairs that are second-order nearest neighbors of one another. For example the arrow from point 1 to point 2 indicates that point 2 is the second nearest neighbor to point 1. The double-headed arrow between points 2 and 4 indicates they are second-order nearest neighbors of one another. Point 2 has the highest leverage since it is the most "connected" by second-order weights.

Nearest neighbour relationships are typically defined among a set of point locations (e.g. geographic locations that are output from the geocoding process) and may be represented as a directed graph or *digraph *(Figure 1). The digraph is a visual representation of the matrix of nearest neighbour weights, **N**. Given a row *i *of matrix **N **we have a 1× *N *vector (**n**_*i*_) whose entries define the *k *nearest neighbors (*k*-NN) of point *i*. For nearest neighbors a given entry may be represented using elements in the range 0 to 1 where 1 indicates location *j *is a *k *NN of location *i*. When nearest neighbors are "tied" so that two points are equidistant from a given location *i*, the weights may be discounted using 1/*m*, where *m *is the number of tied nearest neighbors. Nearest neighbor matrices have specific properties. Typically they have 0 along the diagonals, so that a location is not considered to be a nearest neighbor of itself (e.g. *w*_*ii *_= 0). The row sums of nearest neighbor matrices sum to *k*, the number of nearest neighbors being considered, and the matrices are not necessarily symmetric about the diagonal, since nearest neighbor relationships do not have to be reflexive (e.g. *w*_*ij *_≠ *w*_*ji*_). Nearest neighbor weights are used in case-control and space-time interaction statistics such as Cuzick and Edwards' test [26], Q-statistics [32], Vesta statistics [29], and others.

#### Adjacencies

When working with polygons, adjacency may be quantified to reflect the border relationships between polygon pairs. For example, polygons separated by a common border may be considered to be adjacent, otherwise they are not. Polygon-based adjacencies may consider second and higher-order relationships as well. For example, a second-order adjacency arises when two polygons share borders with a third polygon that lies between them. Polygon-based adjacencies may also account for the length of the common border. Adjacency-based weights arise in the analysis of spatial point distributions when "buffering" is used to evaluate geographic proximity among spatial locations (points). Buffering proceeds by taking a given location, *i*, and then drawing a circle about that location (the buffer). Observations whose locations are within the circle are considered to be adjacent to *i *and assigned a "1", those outside are deemed not adjacent to *i *and are assigned a "0". Adjacency matrices, denoted **A**, have specific properties. When binary adjacencies are used the row sums are the number of locations (polygons or points) that are adjacent to a given location *i*. There usually are 0's along the diagonals, so that a location is not considered to be adjacent to itself (e.g. *w*_*ii *_= 0). Adjacency matrices are symmetric about the diagonal, since adjacency relationships are reflexive (e.g. *w*_*ij *_= *w*_*ji*_). Examples of statistics that employ adjacency relationships include the Knox test [28], local indicators of spatial autocorrelation [33,34], and many others.

#### Distance-based weights

Let the elements of a distance matrix be the Euclidean distances between the points. This is a symmetric *N *× *N *matrix with 0 diagonals. To calculate a spatial weight these distances often are rescaled such that nearby locations have larger values, and weights for point pairs separated by large distances are small. Examples include inverse distance weights, e.g.

(4)

Here *a *is a parameter specifying the distance decay (e.g. *a *= 2 yields inverse distance squared) and *d*_*ij *_is the Euclidean distance separating points *i *and *j*. The weights may be rescaled to sum to 1 (e.g. *w*_*i*• _= 1) and in some instances weights for point-pairs separated by more than a given distance are set to 0. Other specifications of distance-based weights are possible and may be based on distances on networks such as rivers and roads, great circle distances and others. Distance-based weights are used in many spatial statistics including Moran's *I*, Mantel's test [27] and others.

This section developed measures of local and global perturbability to positional error, and demonstrated that perturbability analysis may be extended to broad families of spatial weights, including nearest neighbor, adjacencies and distances. Quantification of perturbability requires knowledge of how the adjacency, nearest neighbor or distance-based weights change under positional error. We evaluate the behavior of local and global perturbability in simulation studies later in this paper.

### Leverage Analysis

The question arises as to how the analyst might use the observed geographic arrangement of locations to identify those that are expected to have the greatest impact on the spatial weights and hence on the results of the spatial analysis. We refer to such points as having high *leverage*. Referring to Equation 1, we wish to identify those locations that contribute the most to the weights in the matrix **W**, and hence to the statistic *S*. A simple measure of local leverage is the absolute value of the row sums plus the column sums of the weight matrix:

(5)

Then the location with the greatest leverage is

(6)

This is the location that contributes the most to the weight matrix and hence is likely to have the largest impact on spatial statistics calculated using that weight matrix.

To summarize, leverage is the absolute value of the row and column sum of the weights matrix itself, while perturbability is the absolute value of the row and column sum of the change matrix.

### The LIGA Scatterplot

Leverage and perturbability can be used to identify those locations that need to be geocoded most accurately, under the assumption that such locations are those that (*i*) have the largest impact on the spatial weight and hence on spatial statistics (have high leverage), and (*ii*) have weights that change the most when positional error is introduced (have high perturbability). Once these locations are known they can be visited with a GPS, or one might use aerial survey techniques to more accurately measure location.

The leverage/perturbability scatterplot (LIGA scatterplot for short) is constructed by plotting leverage on the *x *axis and perturbability on the *y *axis. This scatterplot is then divided into 4 quadrants by adding lines defined by the median of the leverage and the median of the perturbability. One could use the mean depending on one's favored measure of central tendency. We prefer the median since it is robust under asymmetric distributions of values. The four quadrants then contain those locations defined as follows.

#### Quadrant 1 (low perturbability, low leverage)

These locations have little effect on the statistic and positional error does not greatly affect their associated spatial weights.

#### Quadrant 2 (high perturbability, low leverage)

For these locations positional error has a large impact on their spatial weights, but they do not contribute much to the spatial statistic.

#### Quadrant 3 (low perturbability, high leverage)

Positional error doesn't have much of an effect on the spatial weights associated with these locations, which contribute a large amount to the statistic.

#### Quadrant 4 (high perturbability, high leverage)

The weights for these locations change substantially when positional error is introduced and they contribute a great deal to the spatial statistic. These are the locations that would benefit the most from increased positional accuracy.

### Positional Accuracy Modeling and Simulation

We wish to predict the impacts on perturbability of different levels of positional error, noting that positional error itself may vary from one location to another. In order to simulate the error, we first need to have a model of how positional error arises. In geocoding applied studies have shown that mean positional error may vary from 50 meters to more than 600 meters depending on the setting (e.g. rural vs. urban) and study (Table 1). It has been shown that this error scales inversely with population density [15], i.e. rural locations on average have larger errors while addresses in urban locations may average about 50 meters of positional error. In order to model such error, we need an understanding of the underlying distribution of error for the study locations. Often the mean error may not be known, since this requires a validation sample by visiting the actual places of residence with a GPS, or by using secondary information such as locations obtained from high-resolution aerial photography. And in fact, only a handful of studies have quantified observed distributions of geocoding positional error [3,15].

Usually geocoding proceeds by using the street segments from a street map file that records where the segment is located geographically, and that associates a range of street addresses with the segment. Odd addresses occur on one side of the street, and even addresses on the other. In such an arrangement one can impute the location along the street segment using linear interpolation in the address range. An offset from the street center is then used to derive the final imputed set of geographic coordinates for the address in question. Positional error in this process then arises from errors in the underlying street map; violations of the assumption that addresses are a linear function along the street segment (e.g. the frontage of the lots along the street segment is not constant); and to differences in offset from the street centerline (e.g. not all houses are located the same distance from the street). Positional error is also introduced during imputation when addresses are entirely lacking and centroids (e.g. of zipcodes or census tracts) are used instead [35].

For an initial starting point, we assumed that a Gaussian process in two-dimensions underlies the positional error, and hence represent the error with a distribution as shown in each Cartesian direction:

(7)

*P(d) *is the probability density function of distances (*d*) from the geocoded coordinates that a location may occur. Note that we are multiplying a standard normal function by 2 since all distance values must be non-negative. Also, to achieve different mean distances that geocoding positional error misplaces a location, this function can be multiplied by a scaling factor, *c*. This distribution has a cumulative distribution function of:

(8)

This integral (commonly called the error function) does not have an analytical solution but we use a numerical approximation for sampling from it. Using this Gaussian distribution as a model for positional error has the added advantage of simplicity. The function shown in Equation 7 can be modified to have different standard deviations. We characterize our equation solely by the mean geocoding error in a study. When more detailed information regarding the probability distribution of geocoding error is available researchers may choose to use more complicated distributions [15]. For our first experiments we simulated a series of random locations placed across a study area of fixed size. We simulated the positional error by perturbing each location according to the Gaussian process described in Equations 7 and 8. For the number of points we used in our simulations, we found that there was little variation in our measures, as demonstrated by a small standard error and averaging the results from ten simulations of all locations yielded very stable measures. We first explored how nearest neighbor weights, are affected by geocoding error representative of the average errors reported by studies cited earlier.

**Figure 2:**
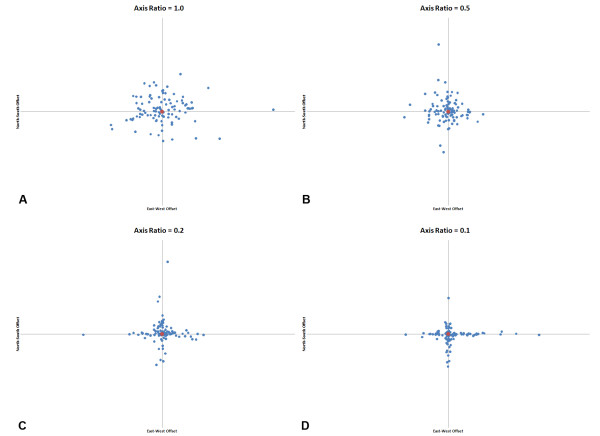
**A "true" location (shown in red) and a sample (100 points in blue) of hypothetical geocoded locations with positional error according to our Gaussian model**. Each figure shows 100 simulated locations, a) for an axis ratio of 1, b) for a ratio of 0.5, c) for a ratio of 0.2, and d) for a ratio of 0.1. The distributions become increasingly cross-like as the ratio departs from 1.

Uncertainty in where to place an address along a given street can lead to positional error being clustered along the east-west or north-south axes [15]. We thus relaxed the assumption of directional independence and examined how distributions that are more skewed towards the east-west and north-south axes affect the patterns of positional error (Figure 2). To do this, we model the positional error as two separate Gaussian functions as in Equation 7, resulting in a "major axis" and a "minor axis" of error. The major axis is in either the east-west or north-south direction (we assume that both are equally likely). Each distribution applies equally to both directions along the axis (i.e. a simulated point has a probability of .5 of lying in each direction along this axis). The major axis has a mean error (1/*λ) *that is larger or equal to that of the minor axis. If the mean errors are equal, then it models directional independence. As the errors diverge, the distribution become more cross-like (Figure 2D).

### Case-control Study of Bladder Cancer in Michigan

We applied the methods to places of residence collected in a study of bladder cancer in south-eastern Michigan in order to identify those four residences that need to be geocoded most accurately. We decided to identify four residences simply to illustrate the LIGA approach - in practice researchers may wish to identify the top 5% or 10%.

For the case-control study, bladder cancer cases, age 80 years or younger upon diagnosis, were recruited from the Michigan State Cancer Registry, and controls were selected from an age-weighted list using a random digit dialling procedure. Controls were frequency matched to cases based on age (± 5 years), race, and gender. Recruitment was limited to individuals who had lived anywhere in one of the eleven counties in the study area (Genesee, Huron, Ingham, Jackson, Lapeer, Livingston, Oakland, Sanilac, Shiawassee, Tuscola, and Washtenaw) for at least 5 consecutive years prior to being contacted. Participants with a history of cancer were excluded, with the exception of non-melanoma skin cancers. All participants were assigned a random identification number to maintain confidentiality. The bladder cancer study was approved by the University of Michigan IRB-Health Committee. Further details on the study design have been published elsewhere [36,37].

## Results

**Figure 3:**
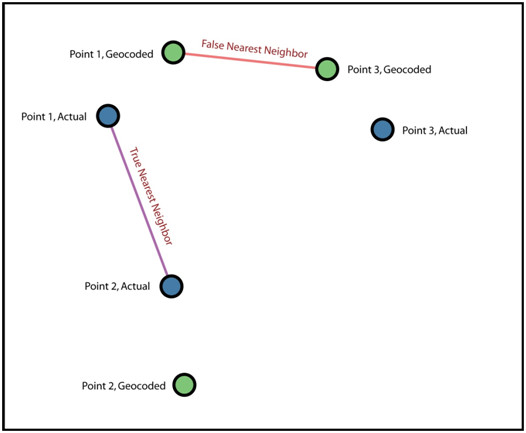
**Geocoding positional error perturbs nearest neighbor relationships by causing an incorrect first nearest neighbor to be found**. The true nearest neighbor for Point 1 is Point 2, but geocoding error makes it appear that Point 3 is the nearest neighbor.

We first consider how sensitive nearest neighbour relationships are to geocoding positional error. To quantify this we measure the number of times positional error caused a change in the spatial weight such that an incorrect first-nearest neighbor was used (Equations 2,3). This process is illustrated in Figure 3.

**Figure 4:**
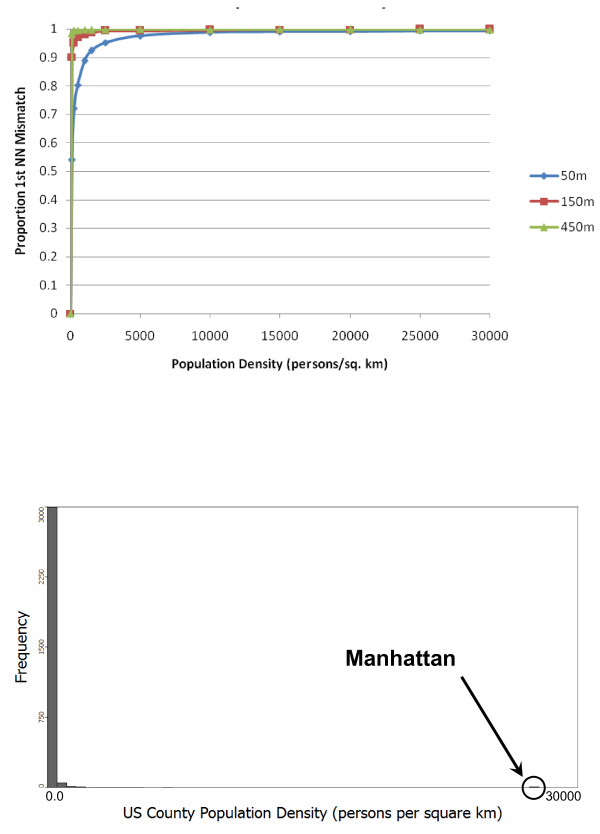
**Global perturbability (proportion of first nearest neighbor mismatch) versus population density (top) and the population density histogram for US counties (bottom)**. All simulations used a population of 500 individuals at the recorded densities. The different lines represent different mean geocoding error distances. The reported values represent a mean from 10 randomizations for each geocoding error/density combination.

We report the global proportion of mismatches of first-nearest neighbors as the measured variable. Other nearest neighbor relationships (e.g. *k*>1) can be measured besides first-nearest neighbors and they follow the same general patterns. Figure 4 illustrates how the proportion of incorrect first-nearest neighbors depends on the density of sampled locations and on the mean positional error when the same error distribution is applied to both axes (a directionally unbiased model).

**Figure 5:**
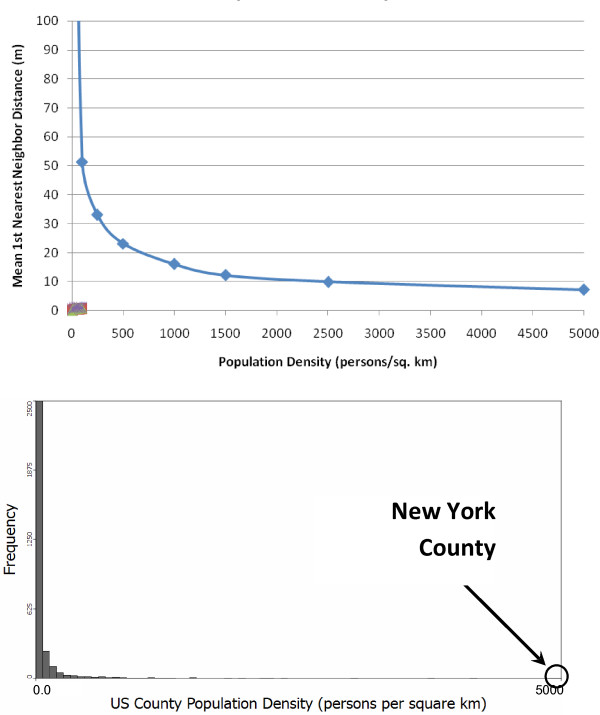
**Mean first nearest neighbor distance vs. population density (top) and population density histogram for US counties (bottom) for population densities up to 5,000 persons per square kilometer**. Results are the mean of 10 simulations of 500 data points in each simulation.

The global perturbabilities shown in Figure 4 are high. In the United States the borough of Manhattan in New York City has an approximate density of 27,500 persons/sq. km, and the state of New Jersey has a density of nearly 440 persons/sq. km - both of which are well over 50% mismatch for all mean geocoding error distances considered. The reason for these extremely high rates can be understood when we examine the relationship of the first-nearest neighbor distance as a function of population density as shown in Figure 5.

The mean first-nearest neighbor distance as a function of density decreases rapidly, reaching a distance of 50 meters (corresponding to the mean geocoding error reported in urban settings) at a density of about 100 persons/sq. km - several orders of magnitude smaller than the density of New York County, which includes Manhattan.

Recall the analysis so far is premised on an underlying spatial Gaussian model so that people occupy random locations. In reality people live in households, and especially in Manhattan, households with locations that only differ in the z-direction (floor of the building). Hence a better measure than population density that would relax the rather unrealistic spatial Gaussian assumption might be residentially-occupied building density.

**Figure 6:**
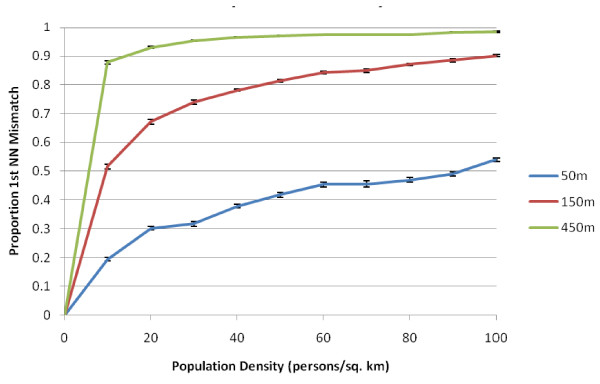
**Global perturbability (proportion of first nearest neighbor mismatch) versus population density at potential study densities**. All simulations used a population of 500 individuals at the recorded densities. The different lines represent different mean geocoding error distances. The reported means and error bars use the means and +/- one standard error from 10 simulations at each geocoding error/density combination.

The simulation model to this point has been used to develop the method and gain insights into how the accuracy metrics behave at population densities typical of US counties. Many studies do not examine the locations of the places of residence of all individuals living within an area, and instead work with a sample from the at-risk population. For example, a case-control study of bladder cancer in Michigan [38] has a density of 6 subjects/sq. km in the urban areas of Ann Arbor and the Detroit metropolitan area. For this reason, we examine the low population density portion of Figure 4 in more detail in Figure 6.

While the global perturbability is still relatively high, at the lower range of population densities the weight matrices may have an acceptably low perturbability depending on the local geocoding positional error and population density of the sample. Notice that a given study sample may have different positional error in different parts of the study area. Geocoding positional error will be higher in rural areas, where population density is lower, and is usually smaller in urban areas. Hence urban areas would use the line corresponding to a small geocoding error (e.g. 50 meters in Figure 6), but would have higher population density than rural areas. This tends to ameliorate the impacts of the increasing functions in Figure 6. Nonetheless, Figure 6 illustrates the importance of routinely assessing perturbability in applied studies.

**Figure 7:**
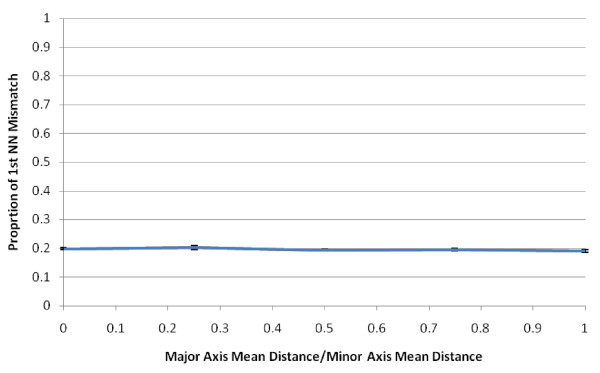
**Changing the shape of the distribution of positional errors does not impact perturbability**. All values represent the mean of 10 randomizations for each major/minor axis combination with confidence intervals of +/- one standard error. All randomizations had a mean geocoding error distance of 50 meters and the sample had a density of 10 persons/sq. km.

Next we examined the effect of the shape of the probability distribution of positional errors on perturbability. For this, we performed a series of simulations with a sample density of 10 persons/sq. km. We relaxed the assumption of using the same probability distribution for both the east-west and north-south axes. For all simulations we kept the same mean geocoding error. A ratio of the *λ*'s of 1 corresponds to a circular distribution and the closer to 0, the more cross like the distribution becomes. As the values of *λ *become increasingly different on the two axes, the distribution becomes cross-like (Figure 2). The shapes of the geocoding error distribution we explored had no effect upon the global perturbability (Figure 7). We believe this result may be extended to a larger family of geocoding error shape models. Specifically, we suggest the perturbability of the nearest neighbor weights depends on the mean positional error, and not on the geographic shape of their probability distribution. However, this generalization needs to be tested with additional error probability distributions before it can be formally accepted.

**Figure 8:**
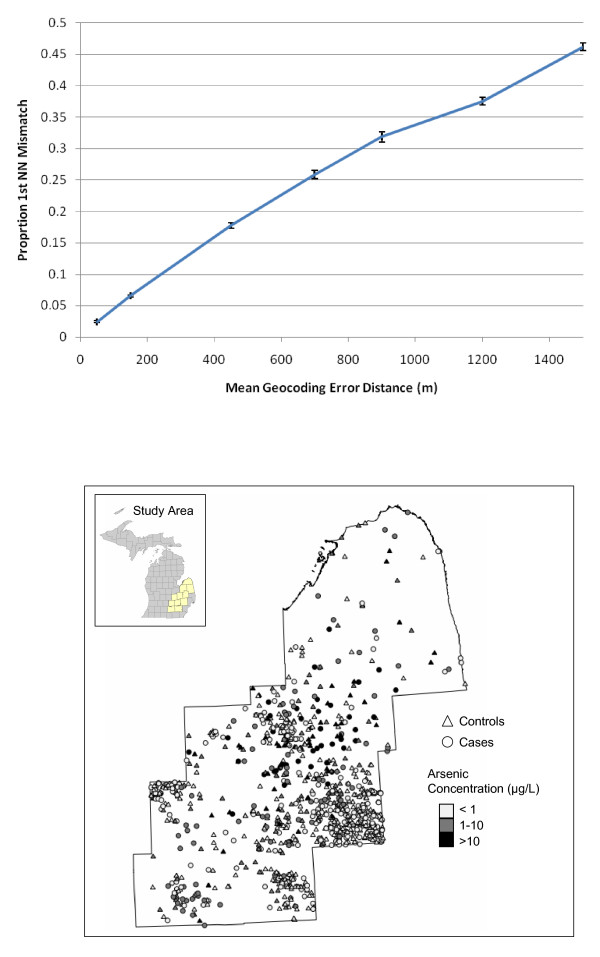
**Proportion mismatch of first nearest neighbors versus mean geocoding error distance (meters) (top) for a study of bladder cancer cases in Michigan (map, bottom)**. Each point represents the mean of 10 simulations on the observed locations (~500 points). The confidence intervals shown are +/- one standard error from 10 randomizations. Bladder cancer map (bottom) from [22], case locations perturbed to protect participant privacy.

We next evaluated perturbability as a function of positional error in an applied study to relax the assumption of homogeneous population density and to demonstrate implementation of our approach. Figure 8 shows the results when we apply our approach to a study of residential locations of cases observed in a study of bladder cancer in Michigan (from [39]). This allows us to assess how sensitive the results of the spatial analyses in these studies might be to different levels of geocoding error. If the geocoding positional error matches the typical values of urban locations (~50 m), the perturbability is relatively low (~.02, 2%), whereas if the geocoding error matches some of the values reported in rural studies (~1500 m), the perturbability is substantial (~.41, 41%).

**Figure 9:**
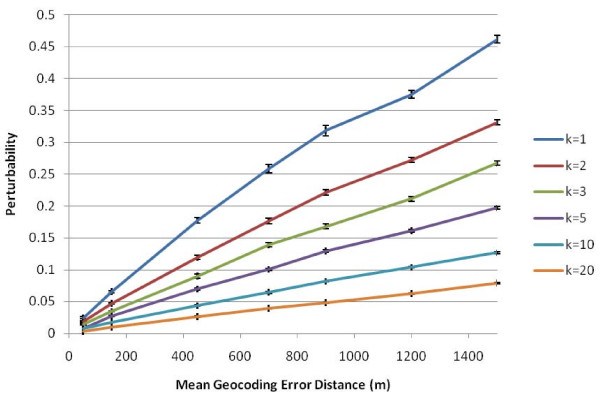
**Global perturbability as a function of mean geocoding error for *k *= 1 to *k *= 20**. The confidence intervals are +/- one standard error from 10 randomizations.

We next considered how the global perturbability varies when using different numbers of *k *nearest neighbors as the spatial weight (Figure 9). When mean positional error is near 600 m, as was reported in rural areas by Cayo and Talbot [5], simulations with *k *= 1 nearest neighbors have perturbability of approximately 22%, those with *k *= 5 have perturbability of 8%, while those with *k *= 20 have perturbability of near 3%. This illustrates that first, the choice of spatial weight specification can dramatically affect perturbability, and second, that weights employing larger kernel sizes appear to be substantially less sensitive to geocoding error. The spatial analyst thus can reduce sensitivity of the results to geocoding error by increasing the size of the spatial kernel function used to calculate the spatial weight, but this must be balanced by the loss of sensitivity to local spatial effects and by the introduction of smoothing error with larger spatial kernels. In addition, the analyst would need to take into account the spatial scale of the process (e.g. clustering) one might reasonably expect in a given study. To balance these countervailing factors in the bladder cancer study when using nearest neighbor based statistics the analyst might choose *k *= 5 or greater assuming geocoding error is 300 meters or less and a perturbability of 5% or smaller.

**Figure 10:**
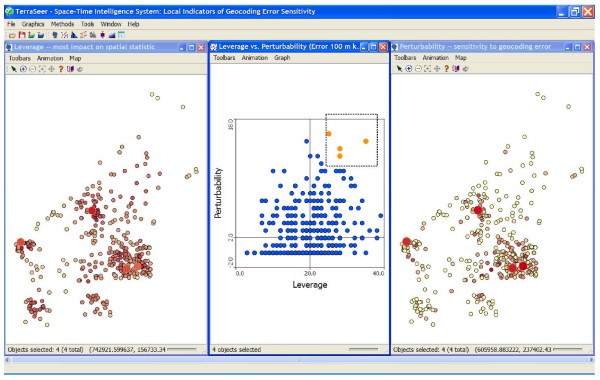
**Application of the Local Indicators of Geocoding Accuracy (LIGA) scatterplot to the bladder cancer case data shown in the bottom of Figure 8**. Those observations in the upper right hand quadrant contribute a great deal to the spatial weights and change the most under a given amount of positional error. The user has brush-selected 4 locations on the scatterplot (center) which are shown by large circles in the maps of leverage (left) and perturbability (right). Additional effort may now be allocated to more accurately determine the locations of those four residences. True locations perturbed to protect participant privacy.

Finally, for the bladder cancer study researchers may wish to identify locations with the highest leverage and perturbability, since these would benefit the most from increased positional accuracy. We used *k *= 20 for the spatial weight and a mean positional error of 100 m. We calculated the local leverage and perturbability for each case and plotted them on a map (Figure 10). Finally, we calculated the LIGA scatterplot and used statistical brushing on the high perturbability - high leverage quadrant to identify those four locations that would benefit the most from additional geocoding effort (Figure 10).

This demonstrates how leverage and perturbability analysis can routinely be used to assess the sensitivity of the results of spatial analyses to positional error, and to identify those locations that need to be geocoded most accurately.

## Discussion and Conclusion

A caveat regarding our approach to analyzing perturbability is warranted. Ideally perturbability would be calculated from a change matrix that is derived from the "true" weight matrix (e.g. **W**), which has been calculated from the "true" locations. We then perturb these true locations under simulation to obtain the weights **W'**. In practice we often may not observe the true locations, and instead analyze weights calculated with an unknown amount of measurement error, that we then further perturb by introducing a known geocoding error under simulation. Is the change matrix we obtain substantially different from the one we would obtain had we been able to use the "true" (but not observed) locations? Yes, it must be different. One property of clustered spatial point distributions is that they become increasingly Poisson as location error is added. Do we expect there to be much of a difference? Given that geocoding error is constrained geographically so that a geocoded address must have certain properties - it must fall within the zipcode, it must be near the street, and so on - we do not believe that "fuzzing" the locations twice (once when observing the locations, and again when we add a simulated error) would greatly change the behavior of the "true" but unobservable change matrix. This conjecture has yet to be verified.

By using a model-based approach of geocoding error, we are able to quantify the expected effects positional accuracy has upon spatial weights and hence on the spatial statistics calculated from those weights. We found that perturbability is directly related to the density of point locations. For this reason, at high densities a given level of geocoding error results in an increased number of incorrect nearest neighbor calculations. At lower densities of points such as might be seen in sampled data, the nearest neighbor distances began to exceed the positional error and the accuracy of nearest neighbor calculations improves dramatically. Herein lies a paradox for spatial analysis: When we increase the sample density to more accurately follow the underlying population distribution, we necessarily increase perturbability and thereby introduce more error into the spatial weights matrix. In some studies the geocoding error may not have significant impacts on the statistical results, and in others it clearly might invalidate the results. In order to have confidence in any given study we therefore must understand the relationship between positional accuracy and the perturbability of the spatial weights.

The results we observed appear to be invariant to changes in the geographical shape of the distribution of positional errors. Our simulations did not incorporate knowledge of the spatial autocorrelation of errors in a place like Manhattan - if 600 Broadway is off by 50 meters to the north, then it is highly likely that the rest of the block is off by the same amount. When available, this information on spatial autocorrelation in positional error can be incorporated into the simulation model. Finally, geocoding error and density of addresses are inversely related and not independent variables. Further studies are needed to better quantify the distributions of positional error.

In our simulations we modelled the distributions of positional error using bivariate Gaussian distributions which can vary in the mean error. Other distributions may better approximate positional error. Further exploration of the variance component was not conducted in our study, but could improve characterization of the positional error distribution. We also conducted our analyses using a model of the positional error distribution that is rhomboidal in shape, but this did not qualitatively alter the findings stated above. In Iowa, Zimmerman [15] found that a mixture of Gaussian or t-distributions provides a good fit. Zandbergen [40] demonstrated that positional errors for GPS locations, geocoded addresses, and LIDAR elevation data may be approximated by Rayleigh, log-normal, and normal distributions respectively, although current standards such as the US National Standard for Spatial Data Accuracy assume the positional error of spatial data is Gaussian, an assumption we employed in our simulations.

The methods developed in this study are broadly applicable for assessing the impact of positional accuracy on the results of spatial statistics and models for any distribution of positional errors, empirical or theoretical. When detailed knowledge regarding positional error distributions is available it can be incorporated directly into our simulation approach. We recommend analysts use the LIGA scatterplot to routinely evaluate the sensitivity of their results to positional accuracy.

## Competing interests

The authors declare that they have no competing interests.

## Authors' contributions

GMJ and RR collaborated on study design and manuscript writing. RR conducted the simulation studies. GMJ finalized the manuscript.
